# Supplementary material for: Peptide-functionalized membrane camouflage for endogenous H2S-induced photothermal immunotherapy of orthotopic colorectal cancer
Source: Nat Commun. 2026 Jan 3;17:168. doi: 10.1038/s41467-025-65876-9 (PMC12780019; doi:10.1038/s41467-025-65876-9)
Supplement: Supplementary file 1 — Supplementary Information File [file 41467_2025_65876_MOESM1_ESM.pdf]

Supplementary Information

Peptide-Functionalized Membrane Camouflage for Endogenous H_2S-Induced Photothermal Immunotherapy of Orthotopic Colorectal Cancer

Kai Cheng [1, 2#], Fang Zhang [1#], Jia-Hua Zou [1, 3, 4#], Xiao-Ling Lei [1], Xiao-Ting Xie [1], Yan-Bin Guo [1], Guo-Ping Wang [1], Bo Liu [1], Yuan-Di Zhao [1*], Jiang Xia [2*], Jin-Xuan Fan [1*]

[1] Britton Chance Center for Biomedical Photonics at Wuhan National Laboratory for Optoelectronics-Hubei Bioinformatics & Molecular Imaging Key Laboratory, Department of Biomedical Engineering, College of Life Science and Technology, Huazhong University of Science and Technology, Wuhan 430074, Hubei, P. R. China

[2] Department of Chemistry, The Chinese University of Hong Kong, Shatin, Hong Kong SAR, P. R. China

[3] Department of Oncology, Huanggang Central Hospital of Yangtze University, No.126 Qi'an Road, Huanggang 438000, Hubei, P. R. China

[4] Hubei Clinical Medical Research Center of Esophageal and Gastric Malignancy, Huanggang City 438021, Hubei, P.R. China

[#] These authors contributed equally to this article.

* Corresponding author. Email address: zydi@mail.hust.edu.cn (Y.-D. Zhao); jiangxia@cuhk.edu.hk (J. Xia); jxfan@hust.edu.cn (J.-X. Fan)

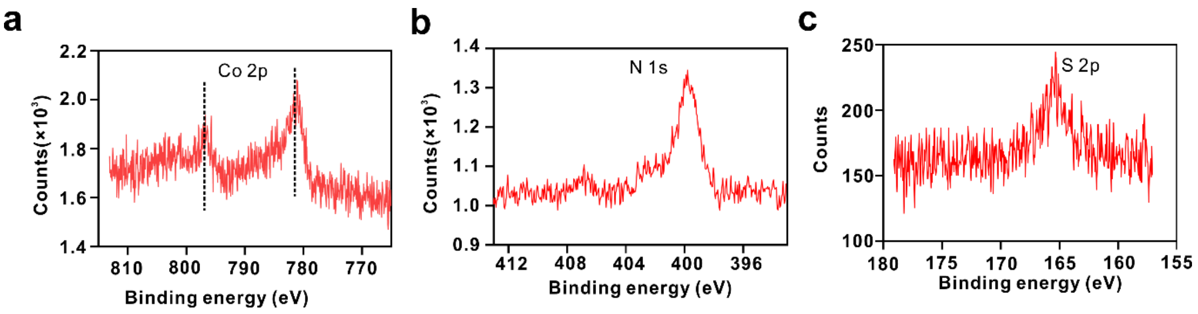

Supplementary Fig. 1. a-c High-resolution X-ray photoelectron spectra of the elements Co, N, and S in PfCC probe.

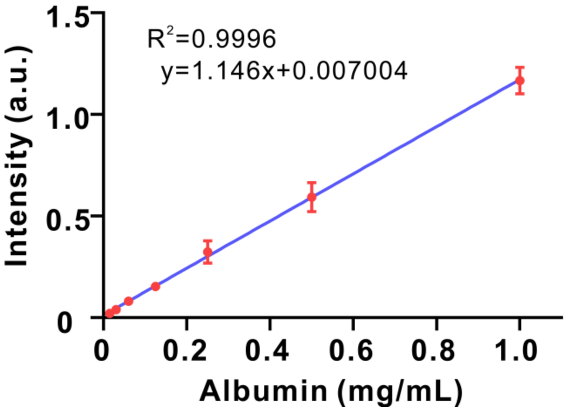

Supplementary Fig. 2. Standard curve of standard albumin concentration versus absorption intensity. Data are expressed as mean ± SD (n = 3 independent samples).

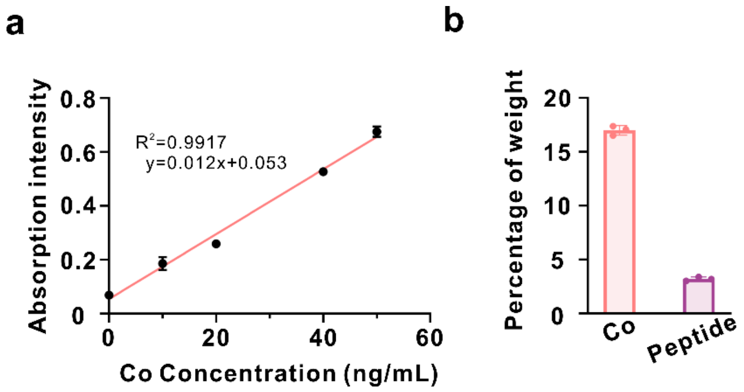

Supplementary Fig. 3. a The standard curve for cobalt concentration and absorption. Data are expressed as mean ± SD (n = 3 independent samples). **b** The proportion of cobalt and antibacterial peptides in the PfCC probe. Data are expressed as mean ± SD (n = 3 independent samples).

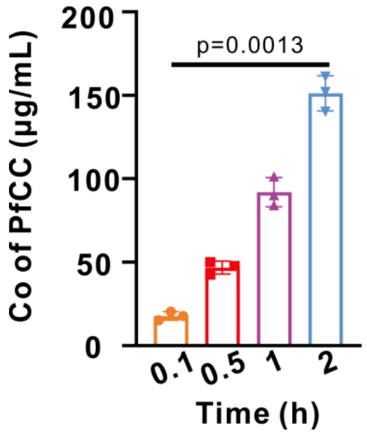

Supplementary Fig. 4. Quantitative analysis of Co during the degradation of PfCC at different times under pH=6.5. Data are expressed as mean ± SD (n = 3 independent samples). Statistical differences were calculated using two-tailed Student's t test, *: $p < 0.05$; **: $p < 0.01$; ***: $p < 0.001$.

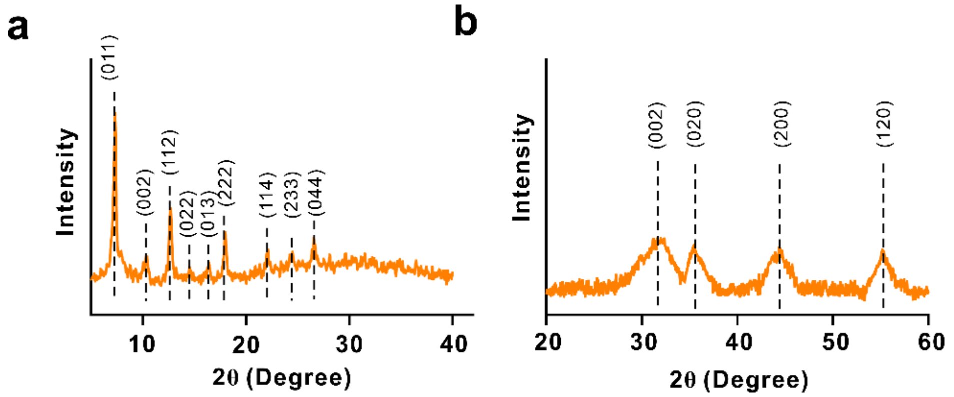

Supplementary Fig. 5. a, b X-ray powder diffraction analysis of Co-MOF and CoS.

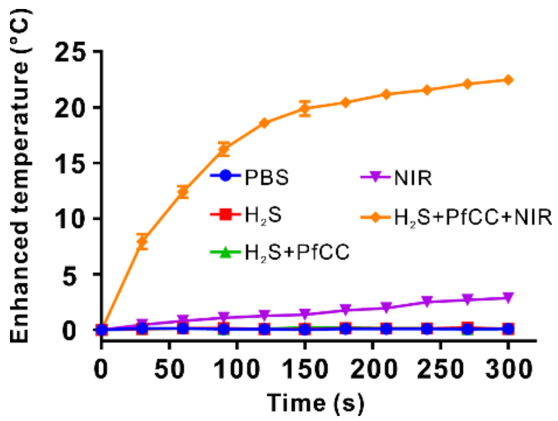

Supplementary Fig. 6. Temperature change of different treatments followed with/without laser treatment at 1.0 W/cm^2. Data are expressed as mean ± SD (n = 3 independent samples).

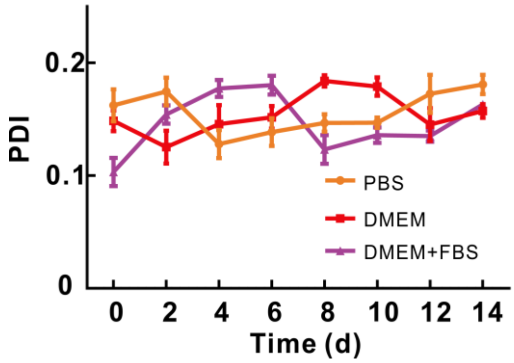

Supplementary Fig. 7. PDI of PfCC in different cultural media with time. Data are expressed as mean ± SD (n = 3 independent samples). PDI: Polydispersity Index.

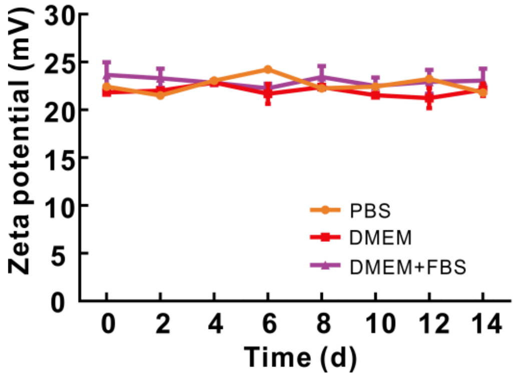

Supplementary Fig. 8. Zeta potential of PfCC in different cultural media with time. Data are expressed as mean ± SD (n = 3 independent samples).

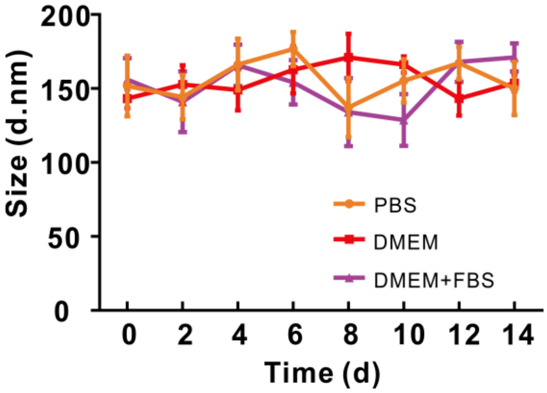

Supplementary Fig. 9. Hydrated particle size of PfCC in different cultural media with time. Data are expressed as mean ± SD (n = 3 independent samples).

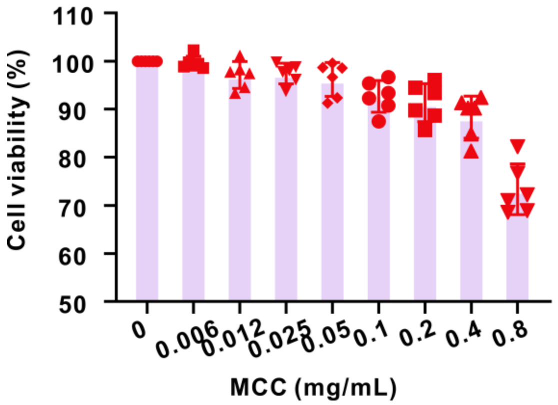

Supplementary Fig. 10. CCK-8 cytotoxicity of 4T1 cells incubated with different concentrations of MCC. Data are expressed as mean ± SD (n = 6 independent samples).

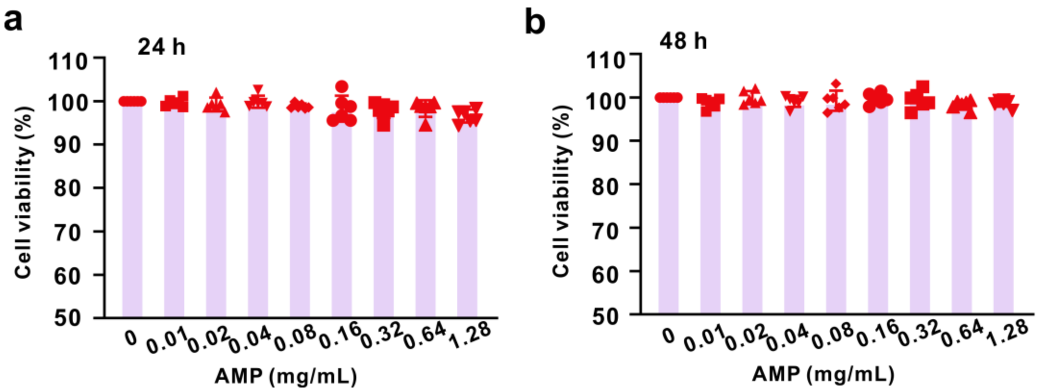

Supplementary Fig. 11. **a, b** Cell viability of CT26 cells after incubation with different concentrations of antimicrobial peptides for 24 and 48 h. Data are expressed as mean ± SD (n = 6 independent samples).

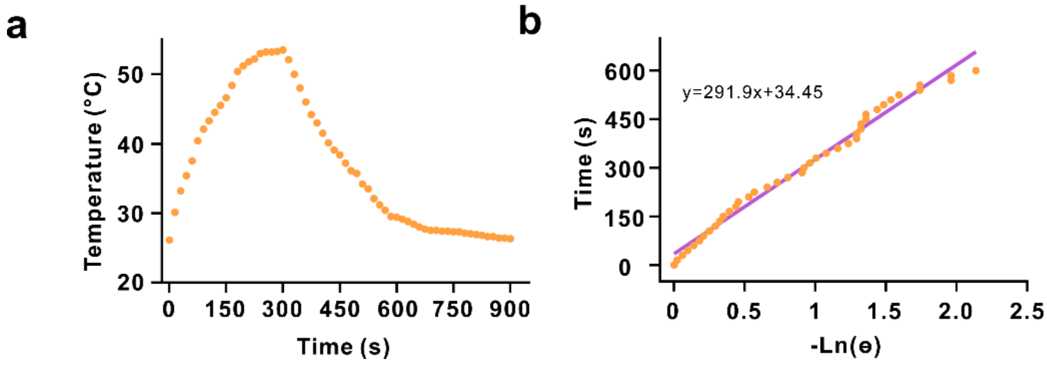

Supplementary Fig. 12. a, b Rise-fall temperature of CoS and calculation of τ value chart. Here, τ represents the constant of the time process of the transition reaction.

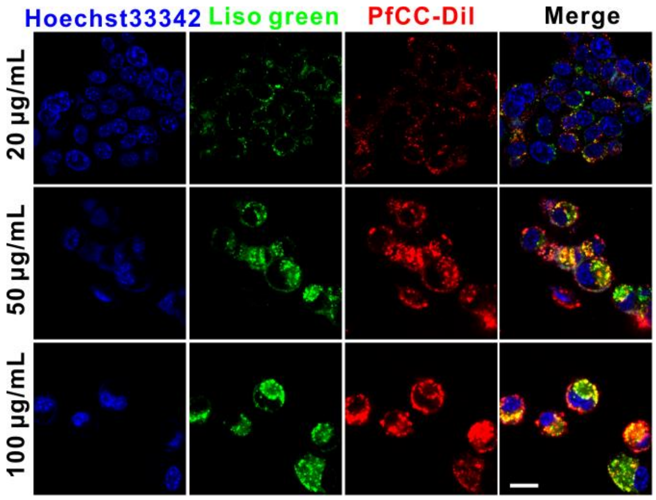

Supplementary Fig. 13. Colocalization imaging of CT26 cells co-incubated with different concentrations of DiI-labeled Co@PM after 4 h. Scale bar: 20 μm. Images are representative of three independent experimental replicates.

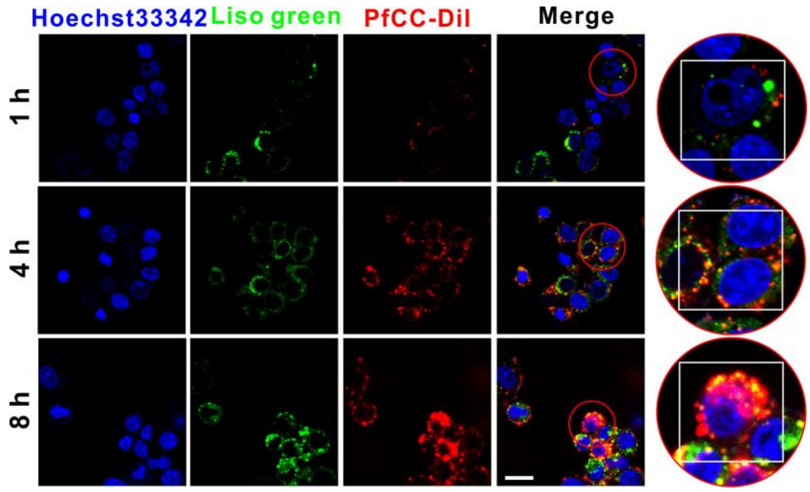

Supplementary Fig. 14. Colocalization imaging of CT26 cells co-incubated with DiI-labeled Co@PM for different time. Images are representative of three independent experimental replicates. Scale bar: 20 μm.

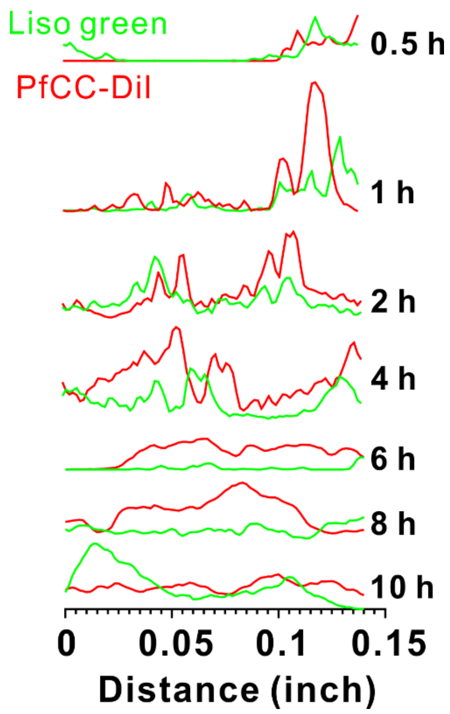

Supplementary Fig. 15. Quantitative analysis of colocalization of lysosomal green and DiI-red fluorescence channel after CT26 cells co-incubated with Dil-labeled Co@PM for different time.

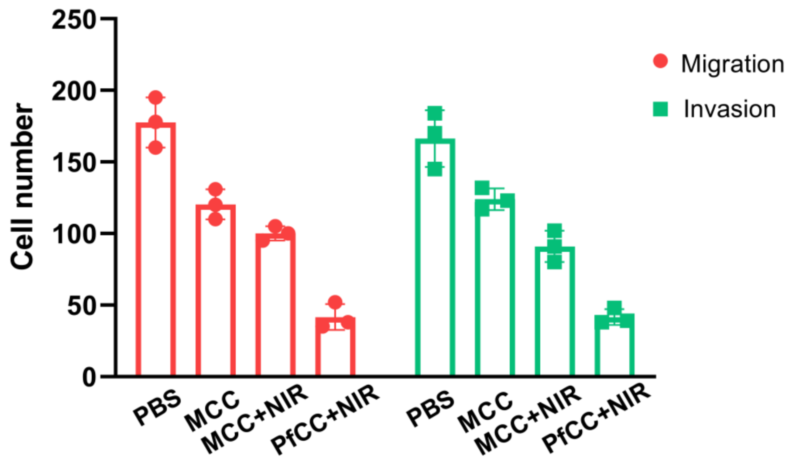

Supplementary Fig. 16. Cell numbers in the lower chamber of the Transwell after incubation and treatment of CT26 cells with different probes. Data are expressed as mean ± SD (n = 3 independent samples).

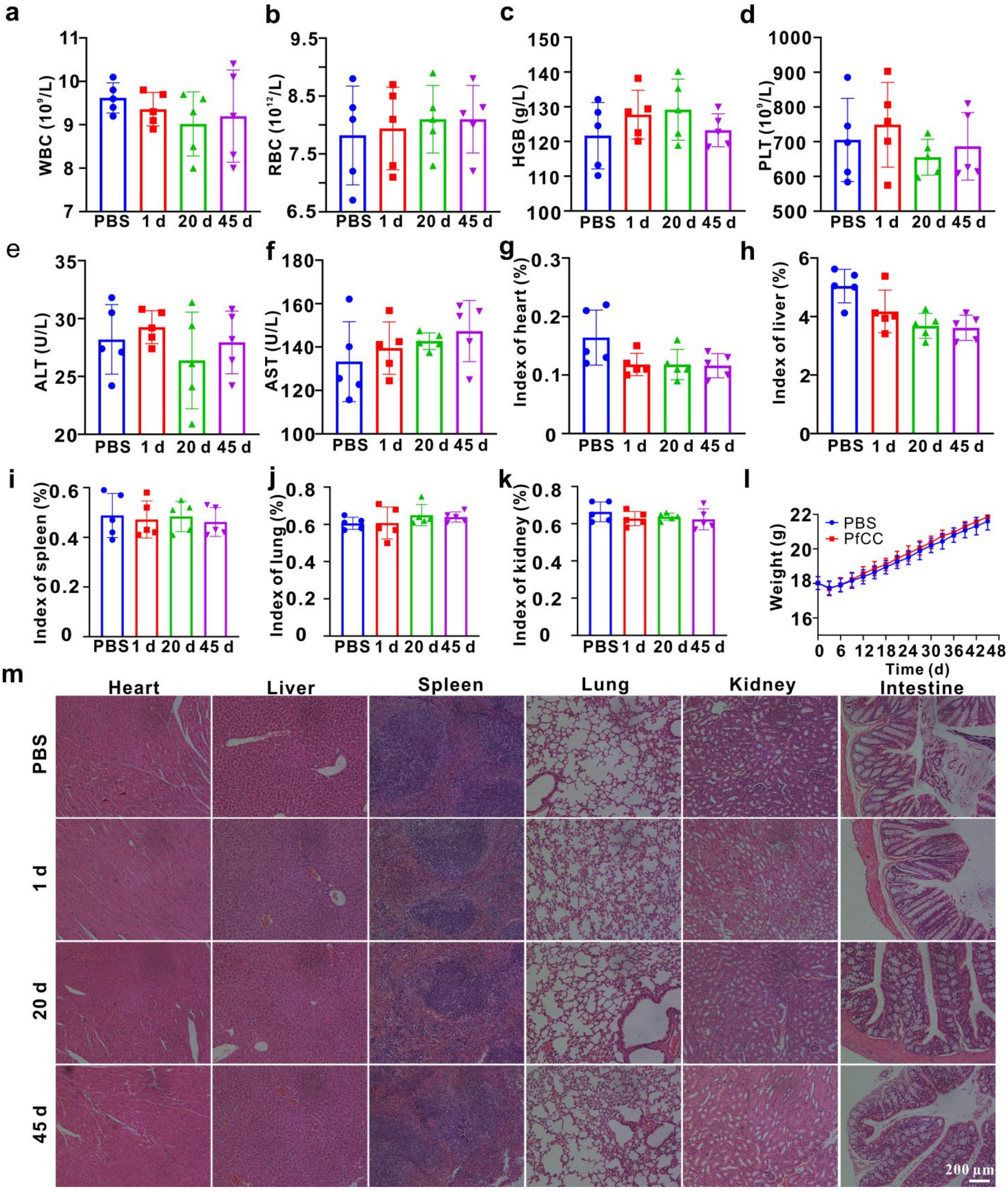

Supplementary Fig. 17. Biosafety of the probe in vivo. Blood analysis of normal mice after Co@PM injection: **a-d** WBC, RBC, HGB, and PLT. **e, f** Analysis of liver function indicators: ALT and AST. **g-k** Corresponding organ index of heart, liver, spleen, lung, and kidney; **l** the change in body weight of mice injected with PBS and probes over 45 d. **m** The HE staining results of the corresponding organs after 45 d of PBS injection, 1, 20, and 45 d of probe. Data are expressed as mean ± SD (n = 5 independent samples). Images are representative of three independent experimental replicates. Scale bar: 200 μm.

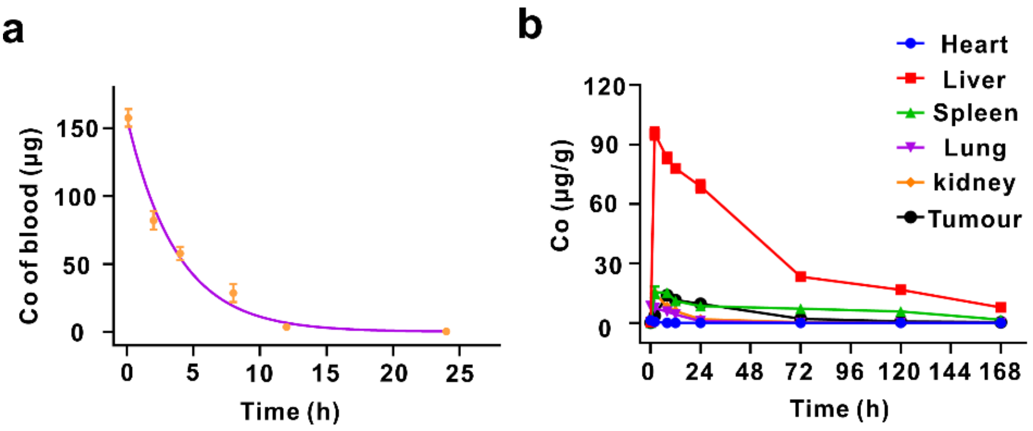

Supplementary Fig. 18. a, b Cobalt ion metabolism of blood, major organs and tumours. The data are presented as the mean ± standard deviation (n = 3 independent samples).

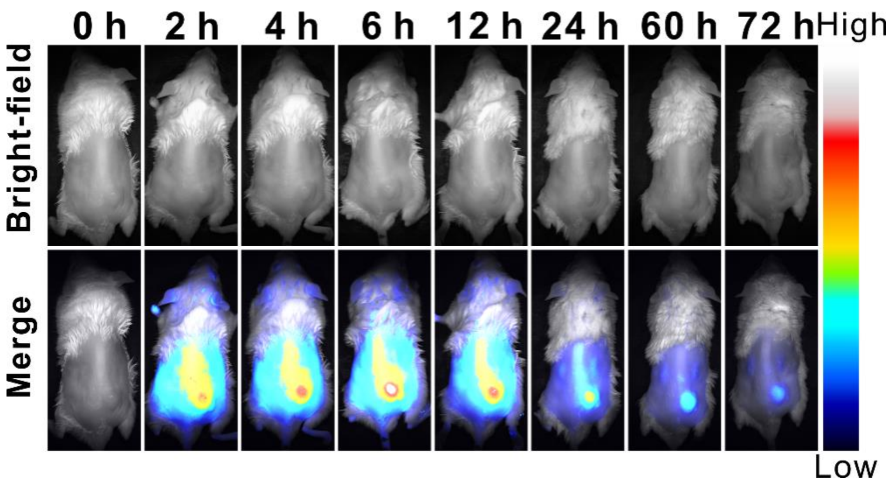

Supplementary Fig. 19. Fluorescence imaging of unilateral subcutaneous CT26 at different time after injection of probes into the tail vein. Images are representative of three independent experimental replicates. Black to white represents the fluorescence intensity values of Cy5.5-labelled PfCC from low to high.

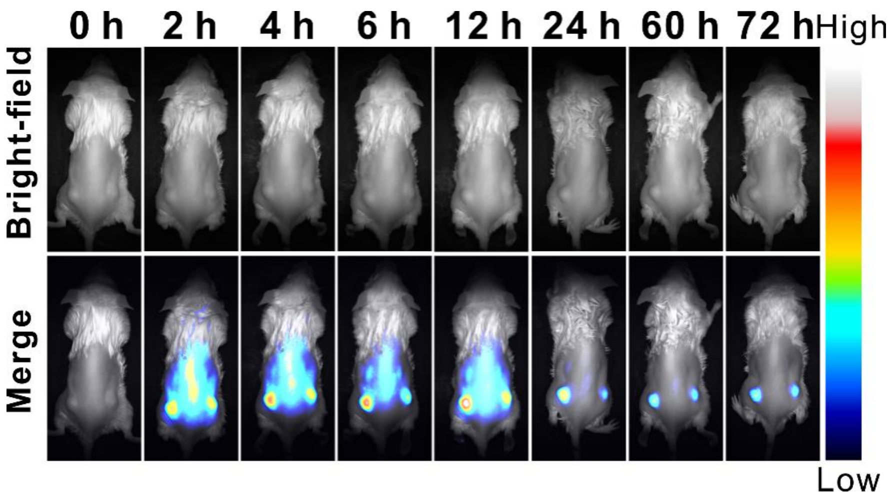

Supplementary Fig. 20. Fluorescence imaging of bilateral subcutaneous CT26 and 4T1 tumours at different time after injection of probes into the tail vein. Images are representative of three independent experimental replicates. Black to white represents the fluorescence intensity values of Cy5.5-labelled PfCC from low to high.

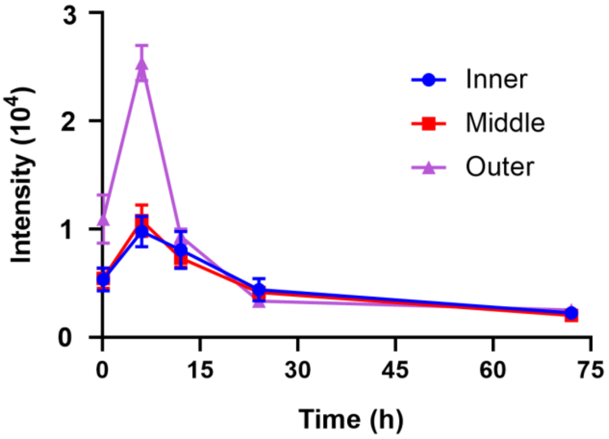

Supplementary Fig. 21. Fluorescence intensity analysis of tumours from inside to outside at different time. The data are presented as the mean ± standard deviation (n = 3 independent samples).

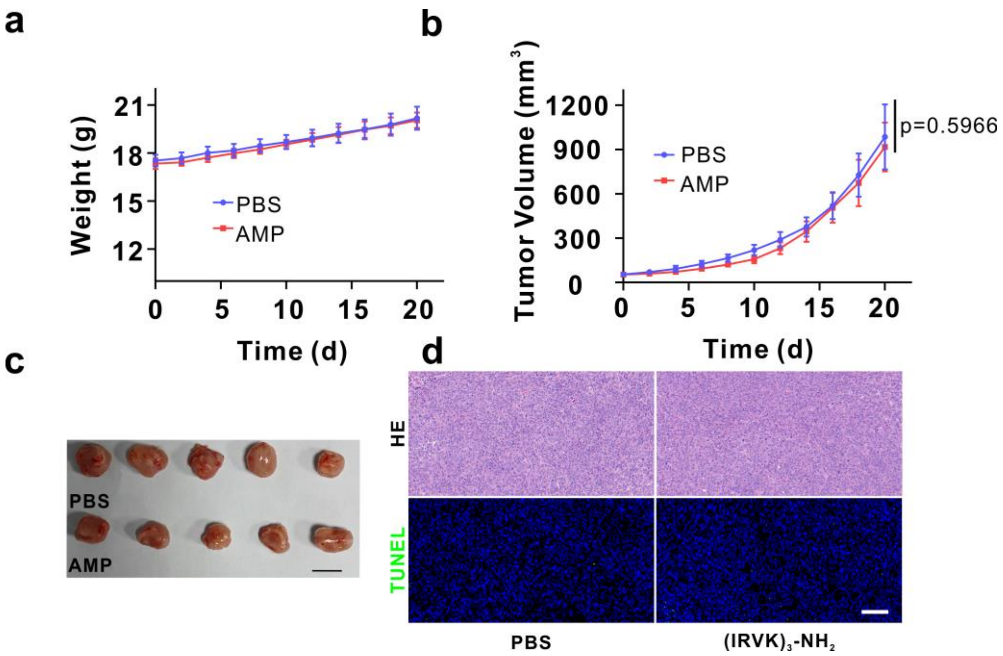

Supplementary Fig. 22. a-c Body weight changes, tumour volume, and tumour bright-field image after different treatments in subcutaneous CT26 tumour bearing mice for 20 d. The data are presented as the mean ± standard deviation (n = 5 independent samples). Statistical differences were calculated using two-tailed Student's t test, *: $p < 0.05$; **: $p < 0.01$; ***: $p < 0.001$. Scale bar: 1 cm. **d** TUNEL and HE staining of tumour sites treated with PBS and AMP after 12 h. Images are representative of three independent experimental replicates. Scale bar: 100 µm.

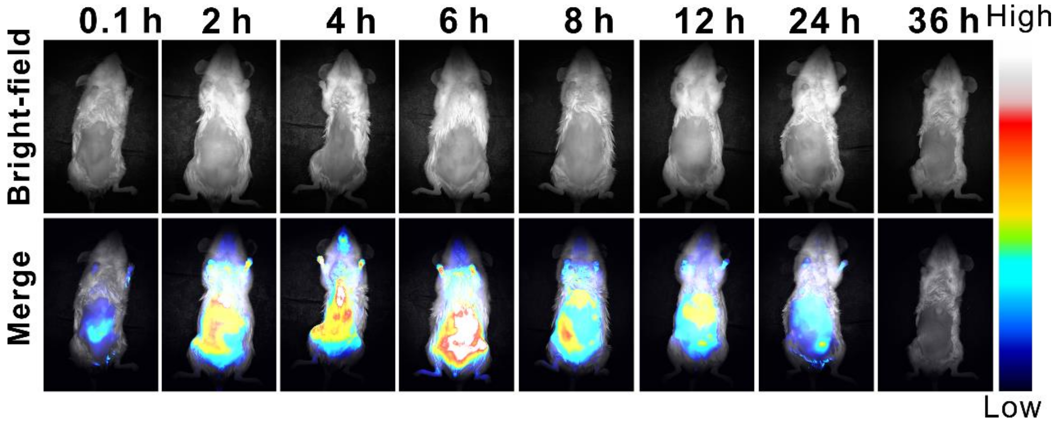

Supplementary Fig. 23. FVB-induced CRC tumours at different time after tail vein injection of probes in mice. Images are representative of three independent experimental replicates. Black to white represents the fluorescence intensity values of Cy5.5-labelled PfCC from low to high.

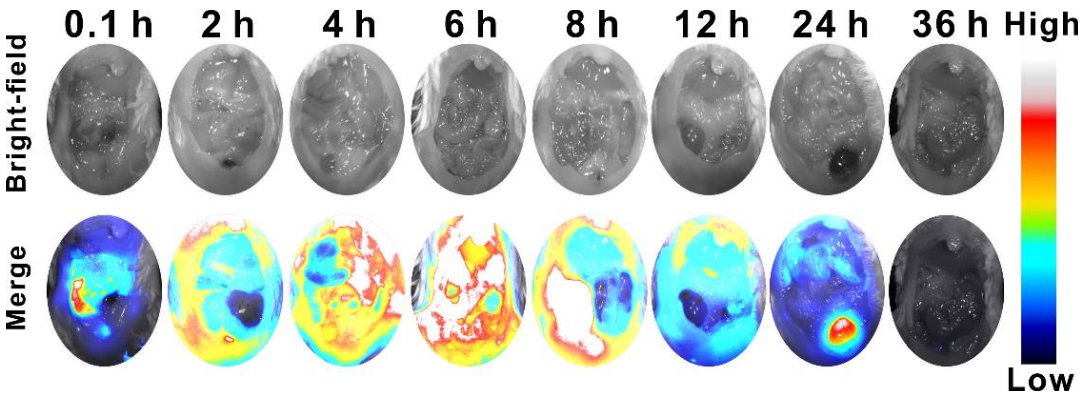

Supplementary Fig. 24. Fluorescence imaging at different time after tail vein injection of probe in FVB-induced CRC tumour mice. Images are representative of three independent experimental replicates. Black to white represents the fluorescence intensity values of Cy5.5-labelled PfCC from low to high.

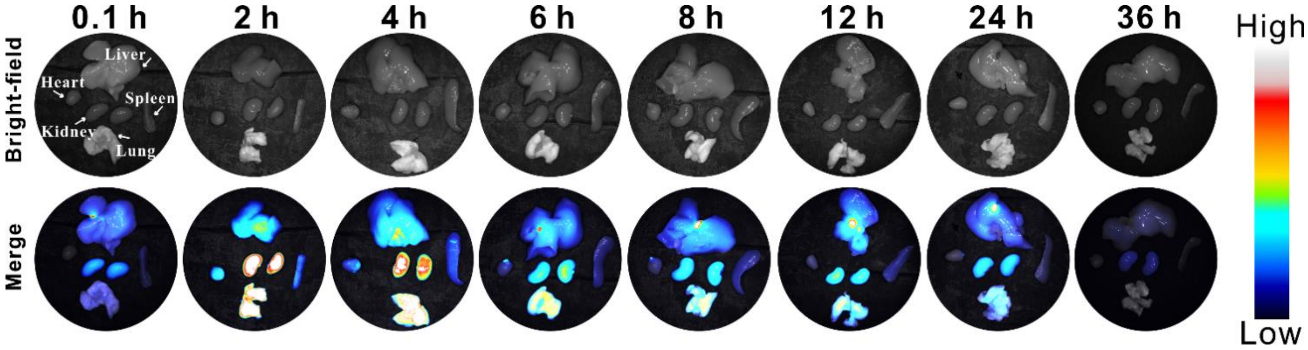

Supplementary Fig. 25. Fluorescent imaging of anatomical organs at different time after injection of probe in FVB-induced CRC tumour mice. Images are representative of three independent experimental replicates. Black to white represents the fluorescence intensity values of Cy5.5-labelled PfCC from low to high.

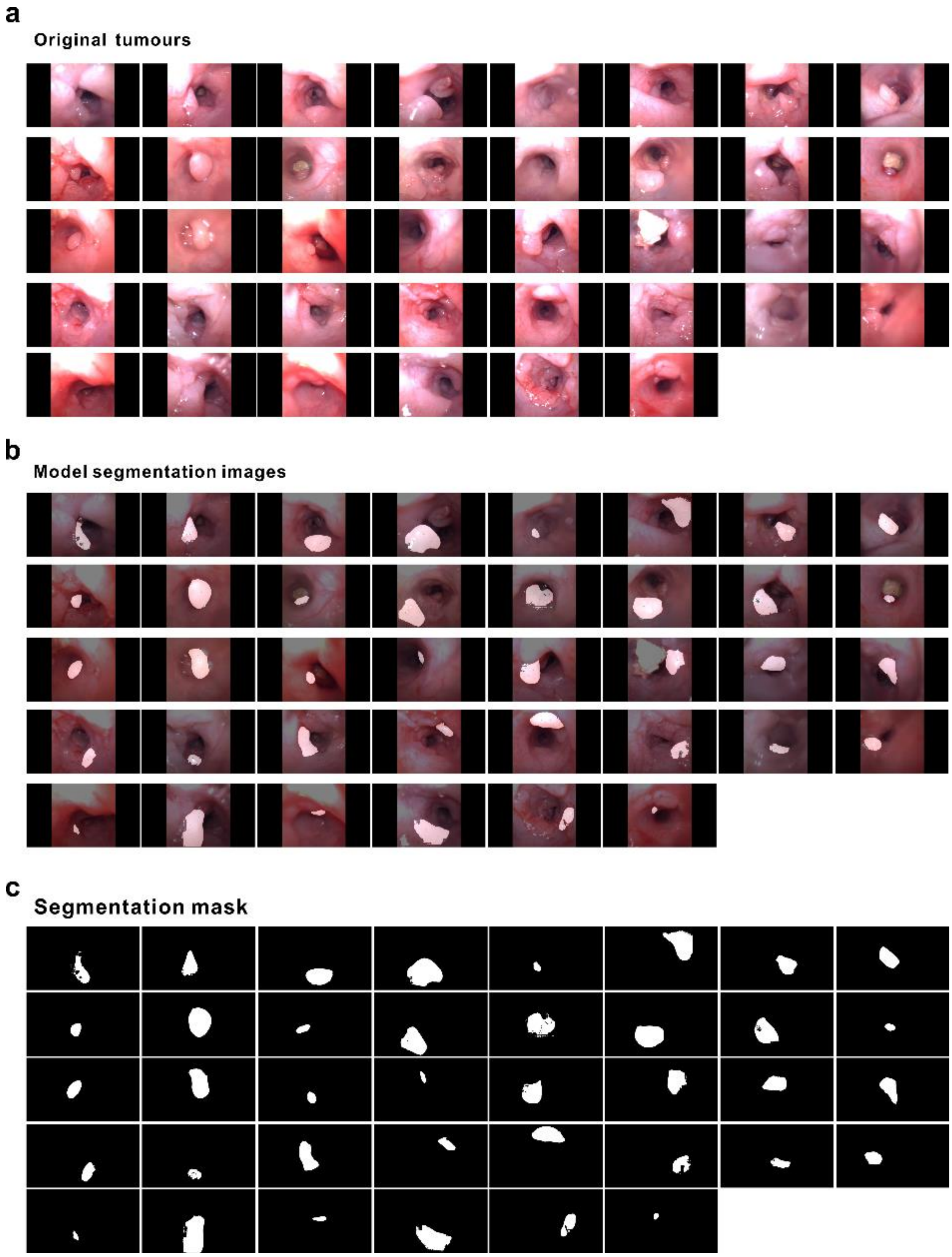

Supplementary Fig. 26. a-c Original tumour, segmented tumour, extracted tumour images based on large model training.

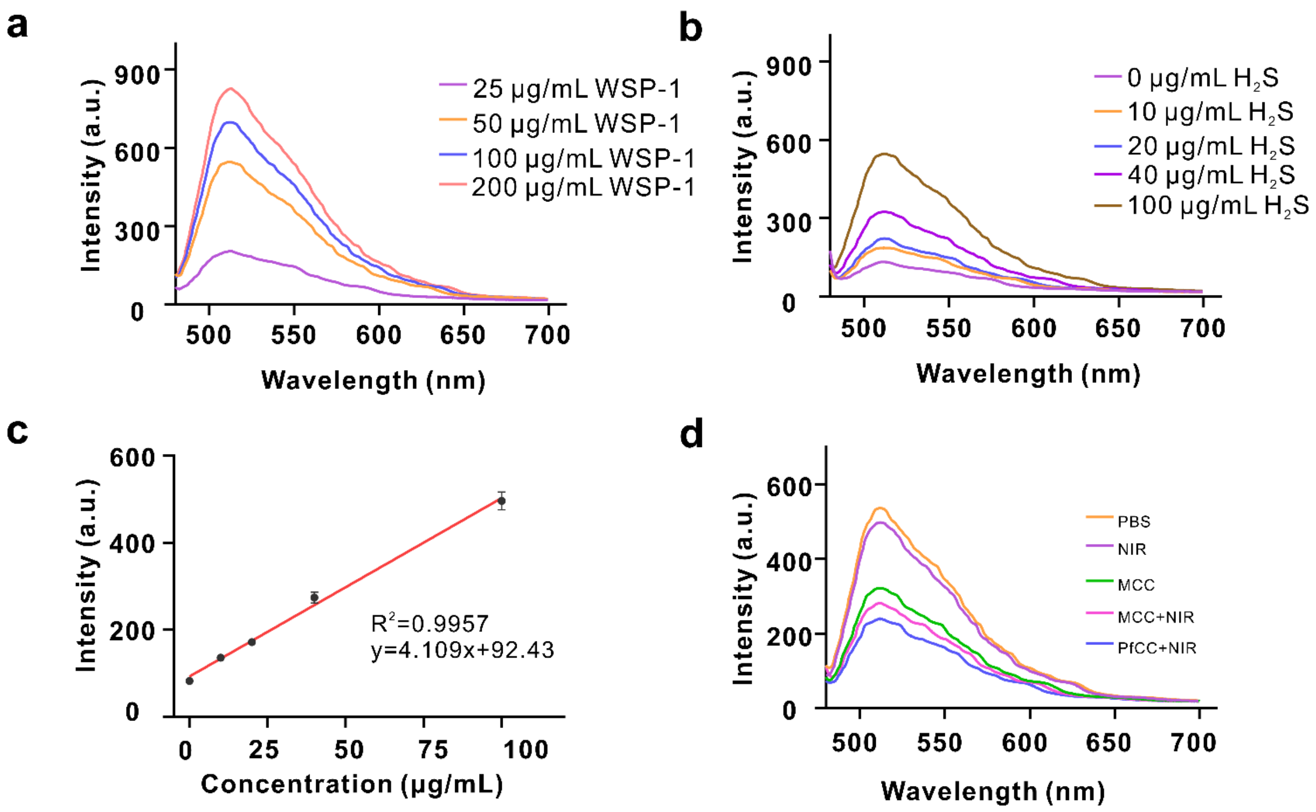

Supplementary Fig. 27. **a** Fluorescence emission spectra of different concentrations of WSP-1 probe reacting with 100 μg/mL of H_2S for 5 mins. **b** Fluorescence emission spectra of 50 μg/mL of WSP-1 probe reacting with different concentrations of H_2S for 5 mins. **c** Standard curve of fluorescence emission intensity at 510 nm and concentration after H_2S reacted with WSP-1 for 5 mins. The data are presented as the mean ± standard deviation (n = 3 independent samples). **d** Fluorescence emission spectra of the extracts obtained from tumour tissues with different treatments after grinding and reacting with WSP-1 for 5 min.

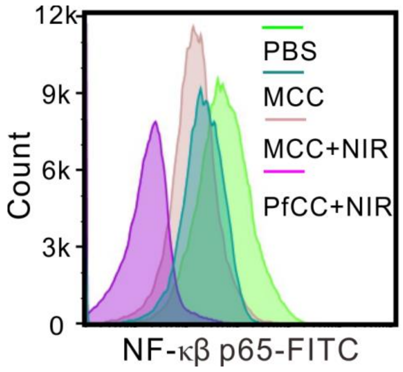

Supplementary Fig. 28. Flow cytometry analysis for the expression of NF-κβ in tumours.

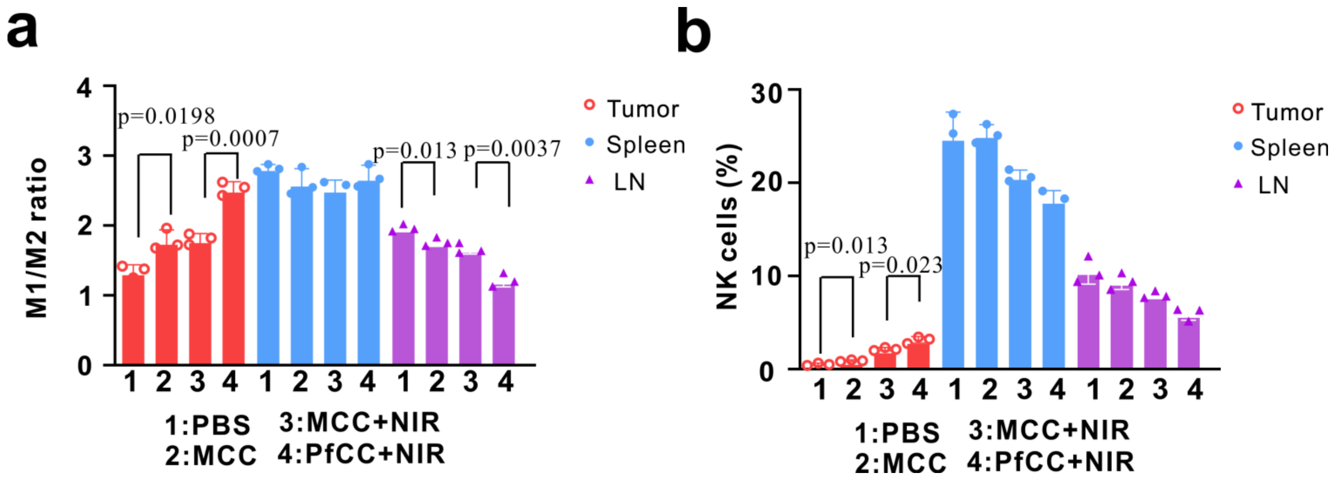

Supplementary Fig. 29. **a, b** Flow cytometry quantification of M1 macrophages/M2 macrophages, and NK cells in tumours, spleen, and lymph nodes. The data are presented as the mean ± standard deviation (n = 3 independent samples). Statistical differences were calculated using two-tailed Student's t test, *: $p < 0.05$; **: $p < 0.01$; ***: $p < 0.001$.

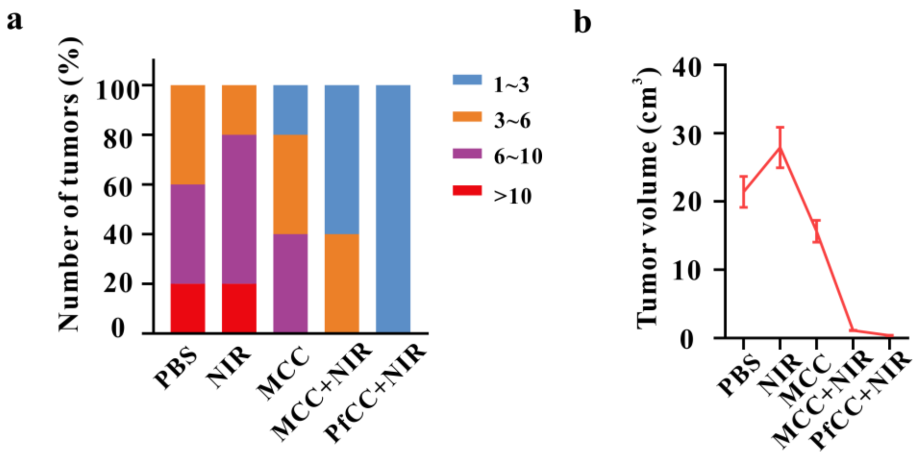

Supplementary Fig. 30. a, b Tumour number and size with in situ induced CRC mice after 6 d of treatment. The data are presented as the mean ± standard deviation (n = 5 independent samples).

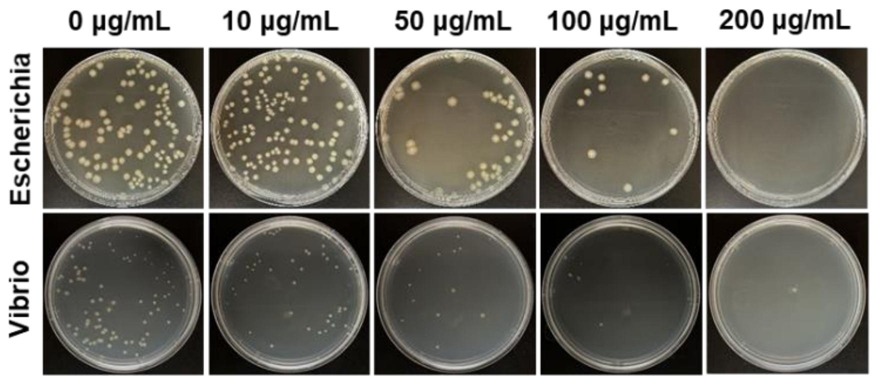

Supplementary Fig. 31. The inhibitory effects of different concentrations of antimicrobial peptides on the growth of *Escherichia* coli and *Vibrio* bacteria. Images are representative of three independent experimental replicates.

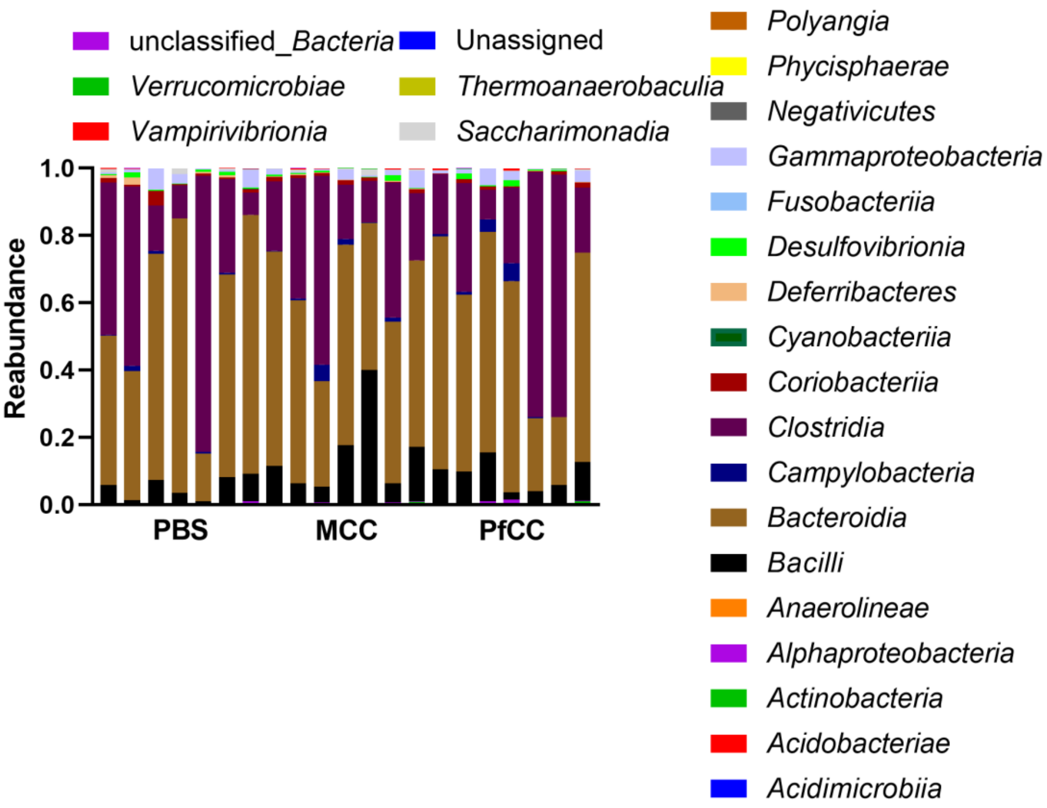

Supplementary Fig. 32. Histogram of the relative abundance of gut microbiota at the class level.

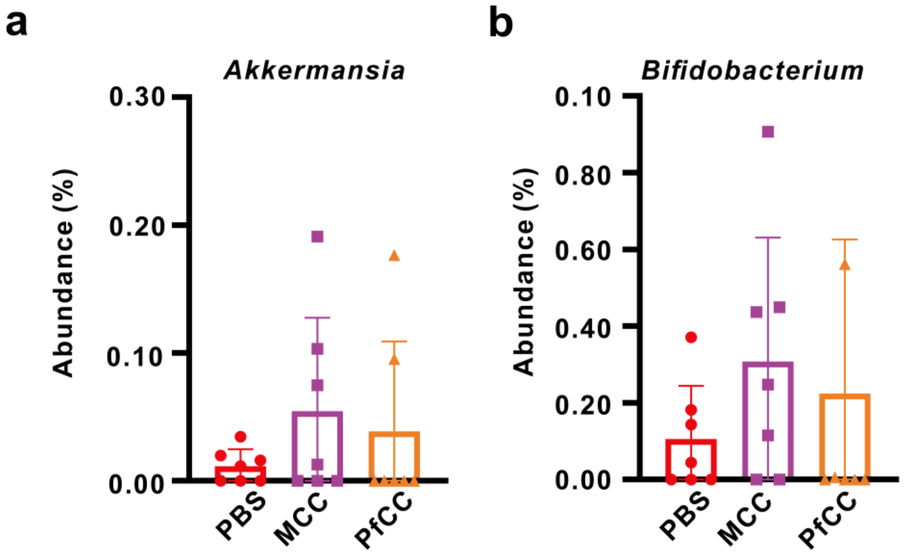

Supplementary Fig. 33. Changes in the relative abundance of *Akkermansia* and *Bifidobacterium* bacteria during 48 h of different probe treatments. The data are presented as the mean ± standard deviation (n = 7 independent samples).
