# Supplementary material for: Peptide-functionalized membrane camouflage for endogenous H2S-induced photothermal immunotherapy of orthotopic colorectal cancer
Source: Nat Commun. 2026 Jan 3;17:168. doi: 10.1038/s41467-025-65876-9 (PMC12780019; doi:10.1038/s41467-025-65876-9)
Supplement: Supplementary file 2 — Reporting Summary [file 41467_2025_65876_MOESM2_ESM.pdf]

nature portfolio

Corresponding author(s): Yuan-Di Zhao

Last updated by author(s): Oct 23, 2025

Reporting Summary

Nature Portfolio wishes to improve the reproducibility of the work that we publish. This form provides structure for consistency and transparency in reporting. For further information on Nature Portfolio policies, see our Editorial Policies and the Editorial Policy Checklist.

Statistics

For all statistical analyses, confirm that the following items are present in the figure legend, table legend, main text, or Methods section.

n/a	Confirmed	
☐	☒	The exact sample size (*n*) for each experimental group/condition, given as a discrete number and unit of measurement
☐	☒	A statement on whether measurements were taken from distinct samples or whether the same sample was measured repeatedly
☐	☒	The statistical test(s) used AND whether they are one- or two-sided<br>*Only common tests should be described solely by name; describe more complex techniques in the Methods section.*
☒	☐	A description of all covariates tested
☒	☐	A description of any assumptions or corrections, such as tests of normality and adjustment for multiple comparisons
☐	☒	A full description of the statistical parameters including central tendency (e.g. means) or other basic estimates (e.g. regression coefficient) AND variation (e.g. standard deviation) or associated estimates of uncertainty (e.g. confidence intervals)
☐	☒	For null hypothesis testing, the test statistic (e.g. *F*, *t*, *r*) with confidence intervals, effect sizes, degrees of freedom and *P* value noted<br>*Give P values as exact values whenever suitable.*
☒	☐	For Bayesian analysis, information on the choice of priors and Markov chain Monte Carlo settings
☒	☐	For hierarchical and complex designs, identification of the appropriate level for tests and full reporting of outcomes
☒	☐	Estimates of effect sizes (e.g. Cohen's *d*, Pearson's *r*), indicating how they were calculated

Our web collection on statistics for biologists contains articles on many of the points above.

Software and code

Policy information about availability of computer code

Data collection	Flow-cytomery data were collected with CytExpert (ver. 2.3.0.84). Confocal images were collected with FluoView31S (ver.2.3.1.163). Animal thermal imaging were collected with Guide Infrared Analysis (ver.1.2.0.0).
Data analysis	Statistical analysis was performed with GraphPad Prism (ver. 8.3.0.538) and Origin 2021(ver. 9.80.200). The three-dimensional images were created with Autodesk 3ds Max (ver. 21.0.845). The plots were assembled with Coredraw x8 (ver. 18.0.0.450). Flow-cytomery data were analyzed with FlowJo VX(ver. 10.0.7.2). ImageJ (ver. 1.4.3.67) were used to analyze fluorescent imaging in vivo. Confocal images were analyzed with FluoView31S (ver.2.3.1.163). AI deep learning environment was constructed with PyCharm (ver. 252.26199.168).

For manuscripts utilizing custom algorithms or software that are central to the research but not yet described in published literature, software must be made available to editors and reviewers. We strongly encourage code deposition in a community repository (e.g. GitHub). See the Nature Portfolio guidelines for submitting code & software for further information.

Data

Policy information about availability of data

All manuscripts must include a data availability statement. This statement should provide the following information, where applicable:

- Accession codes, unique identifiers, or web links for publicly available datasets
- A description of any restrictions on data availability
- For clinical datasets or third party data, please ensure that the statement adheres to our policy

The authors declare that all data generated or analyzed during this study are included in this published article/supplementary Information/Source Data file. The 16S rRNA gene sequencing data generated in this study have been deposited in the Sequence Read Archive under accession number PRJNA1339001. Source data is

available for Fig. 2b-d, Fig. 2f-i, Fig. 3b-f, Fig. 4b-d, Fig. 4f, Fig. 5b-d, Fig. 5h-l, Fig. 6f, Fig. 7b, Fig. 7d, Fig. 7h-j, Fig. 7m-n, Fig. 8b-g, and Supplementary Fig. 1-12, Supplementary Fig. 15-18, Supplementary Fig. 21-22, Supplementary Fig. 27, Supplementary Fig. 29-30, Supplementary Fig. 32-33.

Field-specific reporting

Please select the one below that is the best fit for your research. If you are not sure, read the appropriate sections before making your selection.

☒ Life sciences ☐ Behavioural & social sciences ☐ Ecological, evolutionary & environmental sciences

For a reference copy of the document with all sections, see nature.com/documents/nr-reporting-summary-flat.pdf

Life sciences study design

All studies must disclose on these points even when the disclosure is negative.

Sample size	To ensure the accuracy of the experiments, at least three replicates were performed. In the characterization experiments, 3-4 samples were used to analyze particle size and zeta potentials. In the cell experiments, 3-6 samples were used to analyze biocompatibility, toxicity, and therapeutic effect of probes. In antitumor experiments, 5 mice each group were used to analyze tumor volume, survival rate, body weight in mice. For other experiments, the sample size for each group was 3-5.
Data exclusions	No data were excluded from the analyses.
Replication	The experiments were reproduced successfully,the number of experiments was listed in corresponding figure legends.
Randomization	The samples were allocated into experimental groups randomly.
Blinding	The investigators were blinded to group allocation during data collection and analysis.

Reporting for specific materials, systems and methods

We require information from authors about some types of materials, experimental systems and methods used in many studies. Here, indicate whether each material, system or method listed is relevant to your study. If you are not sure if a list item applies to your research, read the appropriate section before selecting a response.

Materials & experimental systems

n/a	Involved in the study	
☐	☒	Antibodies
☐	☒	Eukaryotic cell lines
☒	☐	Palaeontology and archaeology
☐	☒	Animals and other organisms
☒	☐	Human research participants
☒	☐	Clinical data
☒	☐	Dual use research of concern

Methods

n/a	Involved in the study	
☒	☐	ChIP-seq
☐	☒	Flow cytometry
☒	☐	MRI-based neuroimaging

Antibodies

Antibodies used	FITC anti-mouse CD86 Antibody (BioLegend, Clone:159219, diluted to 1:100);<br>APC anti-mouse F4/80 Antibody (BioLegend, clone:123116, diluted to 1:100)<br>PE Rat anti-Mouse CD49b Antibody(absin, abs1850114, diluted to 1:100)<br>PE anti-mouse CD206 (BioLegend, Cat. No. A17199A, Clone:159219, diluted to 1:100);
Validation	All information about antibodies can be found on the respective antibody website:<br>APC anti-mouse F4/80 Antibody, https://www.biolegend.com/en-us/products/apc-anti-mouse-f4-80-antibody-4071<br>FITC anti-mouse CD86 Antibody, https://www.biolegend.com/en-us/products/fitc-anti-mouse-cd86-antibody-254<br>PE anti-mouse CD206 Antibody https://www.biolegend.com/en-us/products/pe-anti-mouse-cd206-mmr-antibody-7424<br>PE Rat anti-Mouse CD49b Antibody https://www.absin.cn/search.html?keyword=abs1850114

Eukaryotic cell lines

Policy information about cell lines

Cell line source(s)	Mouse breast cancer cells (4T1) and mouse colorectal cancer cells (CT26) were obtained from China Center for Type Culture

Cell line source(s)	Collection (Wuhan, China), mouse colorectal cancer cell-luciferase labelled （Luc-CT26） was obtained Fenghui Biological Technology Co., Ltd.
Authentication	None of the cell lines were authenticated.
Mycoplasma contamination	The cell lines were not tested for mycoplasma contamination.
Commonly misidentified lines (See ICLAC register)	4T1 and CT26 cell lines were of stable origin and there was little possibility of misidentification.

Animals and other organisms

Policy information about studies involving animals; ARRIVE guidelines recommended for reporting animal research

Laboratory animals	7-week-old FVB female mice, BALB/c female nude mice (purchased as 4-week-old, used as 5-week-old, ~18 g), KM male mice (purchased as 5-week-old, used as 6-week-old, ~32 g) were purchased from Beijing Vital River Laboratory Animal Technology Co., Ltd. All mice were housed in a stable environmental conditions (room temperature, 22±1°C, relative humidity, 40-70 % and a 12 h light-dark cycle). All mice had access to food and water ad libitum.
Wild animals	The study did not involve wild animals.
Field-collected samples	The study did not involve samples collected from the field.
Ethics oversight	All animal experiments were approved by the animal experiment ethics committee of Huazhong University of Science and Technology (IACUC Number: 4208).

Note that full information on the approval of the study protocol must also be provided in the manuscript.

Flow Cytometry

Plots

Confirm that:

- [x] The axis labels state the marker and fluorochrome used (e.g. CD4-FITC).
- [x] The axis scales are clearly visible. Include numbers along axes only for bottom left plot of group (a 'group' is an analysis of identical markers).
- [x] All plots are contour plots with outliers or pseudocolor plots.
- [x] A numerical value for number of cells or percentage (with statistics) is provided.

Methodology

Sample preparation	CT26 cells, were derived from mice, and the cells were cultured in a 5% CO2 incubator at 37 °C for 12 h before used.
Instrument	CytoFLEX flow cytometer
Software	CytExpert (ver. 2.3.0.84) was used to collect and analyze the flow cytometry data.
Cell population abundance	After the cells were centrifuged, the pelleted cells were taken for flow cytometric analysis. With adjusting the gain and compensation, 50,000 cells were taken from each group for analysis.
Gating strategy	The untreated cell group was taken, and the voltage was adjusted to make the intensity of FTIC and PI channels around 1000; the single-stained cell group was taken for compensation adjustment and deducting the mutual influence, finally experimental groups were carry out. If the intensity value was greater than 1000, positive signal was considered to exist.

- [x] Tick this box to confirm that a figure exemplifying the gating strategy is provided in the Supplementary Information.
